# The Lateral Calcaneal Lengthening Osteotomy (LCLOT)—A Contemporary Review

**DOI:** 10.3390/jcm14061789

**Published:** 2025-03-07

**Authors:** Ricardo Villar, Simone Santini, Christina Stukenborg-Colsman, Alexandre Leme Godoy-Santos, Victor Valderrabano

**Affiliations:** 1 Arthro Clínica Ortopédica, Recife 50070-170, Brazil; ricardovillar_@hotmail.com; 2 Department of Orthopaedic and Trauma Surgery, Policlinico Universitario Campus Bio-Medico di Roma, 00128 Rome, Italy; s.santini@unicampus.it; 3 Research Unit of Orthopaedic and Trauma Surgery, Università Campus Bio-Medico di Roma, 00128 Rome, Italy; 4 Department for Foot and Ankle Surgery, DIAKOVERE Annastift, Orthopedic Clinic of the Hannover Medical School, 30625 Hannover, Germany; christina.stukenborg@diakovere.de; 5 Laboratório Prof. Manlio Mario Marco Napoli, Faculdade de Medicina, Hospital das Clínicas HC-FMUSP, Universidade de São Paulo, São Paulo 01246-903, Brazil; alexandrelemegodoy@gmail.com; 6 Hospital Israelita Albert Einstein, São Paulo 05652-900, Brazil; 7 Swiss Ortho Center, Swiss Medical Network, Schmerzklinik Basel, 4010 Basel, Switzerland

**Keywords:** foot, ankle, flatfoot, progressive collapsing foot deformity, lateral calcaneal lengthening osteotomy

## Abstract

**Background:** Calcaneal osteotomies are a common procedure in foot and ankle surgery for the treatment of the painful flexible Progressive Collapsing Foot Deformity (PCFD). The lateral calcaneal lengthening osteotomy (LCLOT) allows a three-dimensional foot and ankle flatfoot correction with a single osteotomy. The purpose of this article is to review the types of calcaneal lengthening osteotomies. **Methods:** Review of anatomical, biomechanical and clinical studies and reviews. **Results:** The LCLOT shall be differentiated from the Evans osteotomy or Z-shaped calcaneal lengthening osteotomy. The LCLOT is performed at the sinus tarsi and corrects at the subtalar joint axis biomechanically the pathological hindfoot valgus, foot abduction, and medial arch collapse. The LCLOT technique might vary regarding graft and fixation type. The LCLOT has good clinical results with high union rates. **Conclusions:** The LCLOT is a powerful and successful single-site osteotomy for the triplanar correction of the painful flexible flatfoot/PCFD.

## 1. Introduction

The Progressive Collapsing Foot Deformity (PCFD), historically known as adult acquired flatfoot deformity, is a three-dimensional condition characterized by various degrees of hindfoot valgus, midfoot arch collapse, and forefoot abduction [1]. In the case of associated fore-/midfoot abduction, this often occurs along with talonavicular subluxation [2].

A key contributor to the development of PCFD is the posterior tibial muscle/tendon insufficiency (PTTI) [3]. Beyond its role in plantar flexion, this muscle primarily functions as the primary foot inverter. Its main attachment point is on the navicular tuberosity, with additional attachments to various tarsal and metatarsal structures [4]. The stability of the foot and ankle relies significantly also on the deltoid and spring ligaments. The spring ligament, often implicated in adult-acquired flatfoot, plays a pivotal role by connecting the sustentaculum tali of the calcaneus to the navicular, supporting the talus and therefore the ankle. Its primary function is to provide support to the head of the talus [5]. On the other hand, the deltoid ligament typically becomes affected in the later stages of PCFD. The superficial deltoid ligament has a broad attachment point on the navicular, the spring ligament and the posterior tibiotalar capsule. Its primary role is to counter tibiotalar valgus angulation. The deep deltoid ligament, originating from the intercollicular groove and posterior colliculus, prevents axial rotation of the Talus where it inserts. The deltoid ligament as a whole plays a critical role in supporting the articulating surfaces of the ankle, as well as collaborating with the spring ligament [6]. Therefore, the deltoid and spring ligament are also called the medial ankle ligament complex. This complex insufficiency can lead to medial ankle instability. Chronic instability occurs more commonly after an acute injury failed treatment and may require surgical intervention. In cases where a hindfoot valgus and forefoot abduction is present, deformity correction with a LCLOT is recommended to prevent ligament reconstruction failure [7,8].

Some authors advocated that the presence of an Os Navicularis/Os tibiale externum/Accesory Navicular could lead to posterior tibial tendon insufficiency, and therefore the development of a PCFD [9,10]. This is the most common ossicle in the foot with an estimated incidence of around 14%. It originates from a secondary ossification center of the navicular bone. Although more often asymptomatic, some people develop PCFD symptoms associated with this ossicle, especially those with type 2 (presence of a fibrocartilaginous plate separating from the navicular body).

Tarsal coalition is an anomalous connection between tarsal bones. This connection could be fibrous, cartilaginous, or osseous. Calcaneonavicular and talocalcaneal coalitions represent 90% of the cases and they represent an important diagnosis to consider when facing a child with flatfoot [11]. Also, in adults with a fixed flatfoot/PCFD, a coalition is not rare to be found. After a tarsal coalition resection, an increase of the flatfoot deformity often occurs.

Numerous conservative and surgical treatments have been described for addressing PCFD [12]. Calcaneal osteotomies are common procedures to surgically treat hindfoot/foot malalignment related to this condition [13]. Multiple variations of calcaneal osteotomies have been documented, including techniques like displacement tuberosity osteotomies (medial or lateral sliding calcaneal osteotomies) and lateral calcaneal lengthening (or shortening) osteotomies.

In 1975, Evans described that relative shortening of the lateral column of the foot was associated with calcaneovalgus deformity and with talonavicular subluxation [14]. At that time, he also hypothesized that lengthening the lateral column/calcaneus would correct the flatfoot deformity and described a new technique to correct hindfoot/foot deformities.

Over the years, new techniques were described to lengthen the lateral calcaneal/foot column. The lateral calcaneal lengthening osteotomy (LCLOT) procedure is commonly performed in foot and ankle practice to correct painful and flexible flatfeet deformities.

The goal of this paper is to review the different lateral calcaneal lengthening osteotomy (LCLOT) procedures, fixation methods and graft usage.

## 2. Anatomical Considerations

There are controversies concerning the articular anatomy of the calcaneus and variations between different geographic populations were described [15]. In most cases, three facets (posterior, anterior, medial) are present. In 33 to 78% of the cases, the anterior and medial facets are united either by a narrow bridge or by a continuous articular surface [16]. Functionally, the subtalar joint could be divided into two distinct units each with its own synovial sheaths (the posterior talocalcaneal (TC) unit and the anteromedial talocalcaneonavicular (TCN) unit) separated by the sinus tarsi and canalis [15]. The TCN is also known as the coxa pedis due to its similarity with the hip ball and socket joint [17]. The calcaneal anterior and medial facets belong to this unit. The TCN unit is the part where more ab-/adduction and dorsi/plantarflexion in the hindfoot happens. The TC unit is where most of the inversion and eversion motion of the subtalar joint occurs due to the large posterior talus and calcaneus facets relation [15]. The canalis (posteromedial) and sinus tarsi (anterolateral) delimit an interosseus conic 45 degrees tilted space separating the two units, also called the calcaneus surgical neck. Two major arteries contributing to the subtalar joint vascular supply (A. canalis tarsi and A. sinus tarsi) are in this space [18]. All this complex anatomy is stabilized by a complex ligamentous system [19,20]. It has been suggested that the interosseous talocalcaneal ligament (ITCL) plays a major role in subtalar stability and could also influence ankle stability [21,22], even though the cervical ligament and the extensor retinaculum have a role in stabilizing the joint [23]. Special attention has been given to the deltoid/spring ligament complex that plantarly stabilizes the TCN unit and, when insufficient, could lead to the talonavicular uncoverage seen in PCFD [24].

## 3. Biomechanics

All this complex anatomy implicates complex biomechanics [15,25]. Although considered two distinct functional units, the TC and TCN units do not move independently from one another. It has been described that subtalar motion occurs around a single axis that goes from the lateral aspect of the posterior calcaneal wall towards the center of the navicular and talar head anteromedially [26]. Inversion and eversion, which have been postulated as the most important movement in the subtalar joint, would take place around that axis. The normal range of motion for inversion and eversion is around 40 and 60°, with greater inversion than eversion [27]. Actually, movement at the subtalar joint is triplanar which makes the terms used to describe motion confusing. Inversion, adduction and plantar flexion occur together whereas eversion, abduction and dorsiflexion occur together [25]. An example of the combined motion is the midtarsal joint locking mechanism during gait. On heel strike, during normal kinematics, the heel is in valgus and the midtarsal axis (TCN and calcaneocuboid joint) is parallel to the TC unit which allows more motion on the mid and forefoot, thus permitting weight acceptance. For propulsion, the heel moves into varus and the axis between the two units diverges, diminishing movement in the mid and forefoot and contributing to weight transfer for pushoff [26,28]. Furthermore, tibial rotation has influence on foot position through the ankle and subtalar joint. Internal rotation of the leg pronates the subtalar joint and increases force on the first ray. On the other hand, external rotation of the leg inverts the subtalar joint and increases force on the lateral rays. This concept is known as tibial torque [27,29,30]. Regarding PCFD physiopathology, a valgus subtalar axis stresses the medial soft tissues which go into failure with time contributing to peritalar joint subluxation progression [31,32,33]. Understanding the complexity of the subtalar biomechanics is important for planning the osteotomy as some of them are situated more proximal or more distant from the axis.

## 4. Indications

The main indication for a LCLOT is to treat PCFD in patients with foot abduction (which more commonly occurs at the talonavicular joint). Foot abduction could be clinically assessed by the too many toes sign, where on a posterior view the examiner can see more than one toe laterally. On weight-bearing radiographs, abduction is measured by the talonavicular uncoverage, AP Meary angle (1st metatarsal—talus angle) or the lateral incongruency angle. A recent consensus stated that more than 40% talar head uncoverage at the talonavicular joint would be an indication for a LCLOT procedure [34]. The LCLOT is commonly performed in association with other soft tissues and osseous procedures to address all the three-dimensional abnormalities presented in a PCFD. Common indications are shown in Table 1.

## 5. Definition: Lateral Calcaneal Lengthening Osteotomy (LCLOT) Versus Evans-Osteotomy

**Figure 1:**
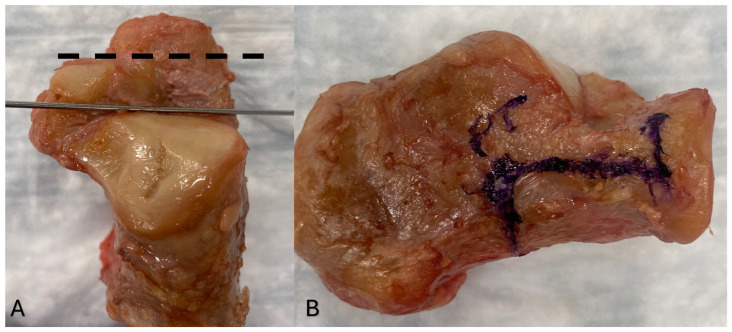
Types of lateral calcaneal open-wedge lengthening osteotomies. (**A**) The Evans Osteotomy (black dashed line) is located more anterior in the calcaneus, 15 mm away from the CC joint and parallel to it. The sinus tarsi osteotomy (LCLOT) is more posterior (delimited by the k wire) and it runs through the interval between the posterior and middle facets. (**B**) Z-shaped osteotomy.

There are three commonly performed osteotomies—for the lateral calcaneal open-wedge lengthening effect—which vary on the site and/or shape of the osteotomy (Figure 1).

1. Anterior (cervical) osteotomy—“Evans-Osteotomy”
2. Sinus tarsi osteotomy—lateral calcaneal lengthening osteotomy LCLOT—“Hintermann-Osteotomy”
3. Z-shaped Lengthening Calcaneal Osteotomy—“Griender-Osteotomy”

As described by Evans, the anterior (cervical) osteotomy was the first one to address the lateral column lengthening concept with a cut 15 mm proximal to the calcaneocuboid (CC) joint and parallel to it [14]. The osteotomy site was filled with a 1 cm tricortical iliac graft. Some variations concerning the distance to the CC joint and its obliquity were described later. Complications with this technique have been well described. Chronic lateral pain, CC joint subluxation and/or arthritis, over/undercorrection, nonunion, graft displacement, sural nerve or peroneus brevis injury, articular surface violation [35]. Lateral pain is one of the most common complications found in 11.2% of the patients [36]. Probably this happens due to plantar overload throughout the whole lateral column and could explain why some patients develop fifth metatarsal stress fractures or even CC joint arthritis [37,38]. Some advocated that performing a distraction arthrodesis of the CC joint would be better to prevent lateral column overload secondary to the Evans procedure [37]. On the other hand, other authors sustain that the CC joint arthrodesis is associated with higher complications and limits hindfoot mobility, which could worsen the lateral overload, while the lateral column lengthening procedure seems to have little implication in subtalar motion [38,39,40]. Due to potential complications with the Evans procedure, other techniques arose and the literature can be confusing with the terms as many surgeons use the term Evans Osteotomy to describe others LCLOT procedures [41,42,43,44].

It is important to consider that many papers use the term Evans osteotomy to describe a LCLOT or sinus tarsi osteotomy. It is important to make that distinction because Evans original osteotomy was situated 15 mm proximal to the CC joint. Using a specific distance to perform the cut means that the osteotomy could be in different positions in relation to the calcaneal articular surfaces, depending on the size of the foot. We recommend using for the sinus tarsi OT our neutral terminology LCLOT, as LCLOT and EvansOT are different in location and in outcomes.

The LCLOT is an osteotomy performed at the “floor” of the sinus tarsi, i.e., more proximally compared to the Evans, at the osseous space between the posterior and middle facets (Gissane angle apex), as described by Hintermann et al. [45]. This osteotomy is performed at the subtalar’s center of rotation, thus it has the theoretical advantage of improving talonavicular coverage with shorter grafts and, since it is performed at the interval between the TC and TCN units, it is associated with less iatrogenic articular damage than the Evans’ osteotomy [46]. Since the latter is performed more distally, it can potentially violate the anterior and/or medial articular facets. Hyer et al. [47] studied 755 calcanei and found that 56.03% had a conjoined anterior and middle facet and that those who had separate anterior and middle facets presented a mean width separation between them of only 3.85 mm which would be a narrow safe zone for the osteotomy and this minority of the calcanei studied. This study also found that the mean distance from the CC joint to the posterior margin of the anterior facet averaged 11.04 mm, which indicates that the Evans procedure would violate the articular surface [47]. More recently, Ettinger and coworkers [46] performed these two osteotomies on 14 cadaveric feet and compared both regarding the damage to anatomic structures. They found that the calcaneal anterior and medial articular facets were intact after the LCLOT/Sinus tarsi Osteotomy in 100% and 85.7% and after the Evans procedure in 42.9% and 71.4%, respectively. The posterior articular surface was unaffected in any cadaver [46]. Ettinger et al. [48] clinically compared these two osteotomies retrospectively, and, although no statistical difference was found in the clinical and radiographic evaluation, they suggested that the LCLOT/Sinus tarsi Osteotomy could lead to fewer degenerative changes at the CC joint.

The Z-shaped Lengthening Calcaneal Osteotomy, also called step-cut osteotomy, requires two wedge grafts and consists of two vertical osteotomies (one-half dorsal and distal and the other one-half plantar and proximal) connected by a transverse long osteotomy. The distal cut is made 8 to 12 mm from the CC joint and the proximal cut is at the level of the peroneal tubercle [49]. Potential theoretical benefits of this technique would be a greater improvement in valgus correction while maintaining the abductus correction with a lower complication rate. Saunders et al. [50] retrospectively compared 143 feet (65 Evans, 78 Z-shaped) with minimum two years follow-up and found faster healing times and smaller graft size in the Z osteotomy group. No differences were found regarding clinical scores or lateral column pain. Demetracopoulos et al. [51] retrospectively reviewed 37 consecutive patients with two years minimum follow-up and found good talonavicular coverage improvement and functional results with no incidence of delayed union, nonunion or graft collapse. Studies comparing the amount of hindfoot valgus correction with sinus tarsi osteotomy and with step cut osteotomy are lacking. Some variations were also described for this technique [52].

## 6. Graft

Traditionally, autologous iliac tricortical grafts have been used to lengthen the lateral calcaneal open wedge osteotomy, but allograft and metallic wedges are presented as alternative options with potentially less morbidity (e.g., donor site pain) and apparently similar results [53,54,55,56,57,58]. Allografts have been used with or without biologics, which could theoretically enhance graft incorporation, although this was refuted by Vosseler in his series [55]. There is no consensus on whether there is a superiority between the options [34].

Dolan et al. prospectively followed 33 randomized feet treated with iliac autograft or allograft and all patients achieved union [53]. Vosseler et al. reviewed 126 LCLOT procedures performed and found no statistically significant difference in loss of correction or nonunion between autograft and allograft [55]. Some studies suggest that allografts were associated with a higher risk of infection and higher costs [54]. Gross et al. retrospectively studied 28 feet that received an LCLOT procedure with a porous titanium wedge and found good pain relief and deformity correction with a high union rate [59]. An open wedge plate can also be used associated with cancellous autograft with apparently similar results [60].

Ideal graft/wedge size has also been discussed. Although graft size is strongly correlated with abduction correction, it has been suggested that the ideal size of the graft should be between 8–12 mm to avoid overcorrection and lateral overload [61]. Larger grafts could also take longer to unite.

Wu and colleagues found recently that amount of correction is improved with graft shape and size (rectangular and larger grafts can provide higher correction degree), but this could affect the contact pressures on the ligaments and joints [57].

Ellis suggested that using templates intraoperatively to estimate talonavicular coverage correction and testing eversion stiffness after wedge insertion could diminish the lateral pain incidence. In his study, this simple measure reduced the incidence of lateral pain (although with no statistically difference) and the revision rates [36]. Kim concluded in a cadaveric study that increasing wedge size correlated with decreasing passive hindfoot eversion and increasing lateral plantar pressure, suggesting that intraoperative preservation of eversion motion may be important for preventing excessive lateral loading [62].

## 7. Methods of Osteotomy Fixation

In Evans’ first description of the osteotomy, no fixation was used. Until today, variations regarding the need and type of fixation exist. Preserving the calcaneal medial wall (lateral open wedge) could enhance osteotomy stability, thus dispensing fixation. Stabilizing the osteotomy with screws or plates prevents graft dislocation and enhances stability, favoring union rates [14,35]. Dayton et al. [63], comparing the calcaneal length after an Evans procedure in patients that had received no fixation or had the osteotomy fixed with a bridge plate, found that plate fixation preserved the correction more than the no fixation method, thus suggesting that fixation could help maintain the correction gained intraoperatively. There is a lack of evidence to suggest any type of fixation method, but it seems that using one screw from distal-lateral to proximal-medial across the LCLOT is the most common method surgeons use. However, plate fixation might have the strongest correction control, e.g., in obesity, osteoporosis, large corrections.

In one of the largest series, Saunders et al. [50] in his series with 143 feet used screws in all patients, being the most common the two screws configuration with one longitudinal screw from the anterior process of the calcaneus going posteriorly and other screw from dorsal to plantar through the sinus tarsi.

Philbin et al. retrospectively evaluated 28 feet treated with a cervical osteotomy fixed with a plate and found only one nonunion (average time for union was 10.06 weeks) and four hardware removals on a 9-month mean follow-up [64].

Foster et al. retrospectively evaluated clinically and radiographically 52 feet, treated with tricortical allograft with screws (26 patients) or opening wedge plate with cancellous chips plus bone marrow aspirate (26 patients) and suggested that the interposition plating technique could lead to lower necessity of hardware removal and nonunion rate [60].

## 8. LCLOT Complications

Although considered a safe procedure, potential pitfalls and complications should always be kept in mind. No objective relation between type of osteotomy, graft size/shape and the amount of triplanar correction obtained was established. So, the literature does not really guide about what to expect about talonavicular coverage improvement, first ray plantarflexion and hindfoot valgus correction with this type of osteotomy; therefore, undercorrection and overcorrection should be a concern while treating these deformities.

Preoperatively, it’s advised to look for signs of associated CC joint arthritis, since it has been described predominantly in the Evans osteotomy that lengthening the lateral column could increase pressure in this joint, hence increasing degeneration speed and symptoms [37]. In such secondary CC joint arthritic cases, it seems that CC arthrodesis is an option, even though some studies have shown suboptimal functional results with this procedure when compared to calcaneal osteotomies, so one should carefully select patients [65,66]. Although a joint sacrificing procedure, it seems that overall hindfoot movement is maintained after CC joint arthrodesis [67,68].

Lateral column pain could arise even in patients with no preoperative signs of CC arthritis. This is probably directly linked to the amount of correction obtained intraoperatively, which makes this an important surgery step to address, and also due to the adaptation of the lateral foot to the new load pattern [69]. The patient should be informed that lateral pain could occur in up to 14.7% of the cases and hardware removal could be necessary in up to 30% [36,70].

Nonunion (especially in elderly, active smokers and osteopenic patients), graft displacement, sural nerve, peroneus brevis injury, articular surface violation, CPRS and infection are also potential complications [35].

## 9. LCLOT Surgical Technique: Authors’ Experience

**Figure 2:**
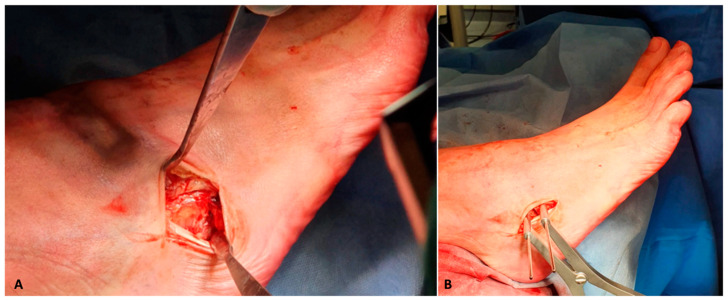
Sinus tarsi approach for the performance of the lateral calcaneal lengthening osteotomy (LCLOT). Note that the plantar Hohmann retractor protects the peroneal tendons (**A**). After the osteotomy is performed with an oscillating sawblade, it’s opened with the help of a K-wire spreader to the necessary triplanar foot correction (**B**).

**Figure 3:**
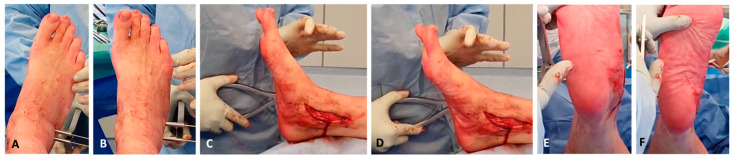
Lateral calcaneal lengthening osteotomy (LCLOT): amount of three-planar correction. Correction of abduction ((**A**) before and (**B**) with LCLOT effect: adduction), correction of medial arch collapse ((**C**) before and (**D**) with LCLOT effect: higher arch), and correction of hindfoot valgus ((**E**) before, (**F**) with LCLOT effect: varisation).

**Figure 4:**
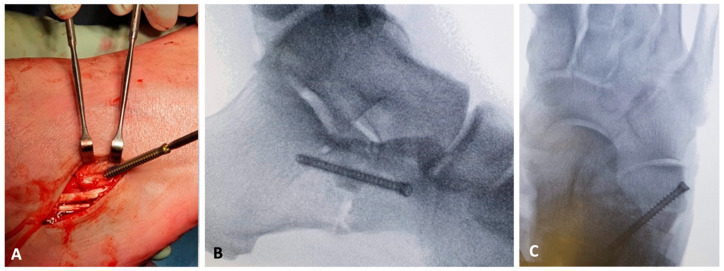
Lateral calcaneal lengthening osteotomy (LCLOT): fixation method. The graft is held in position with a screw (in this case with a CCS 4.0 mm screw, Medartis, Basel, Switzerland) (**A**). Dorsoplantar (**B**) and lateral (**C**) X-ray views show the correct position of the screw, the graft, and the bone.

**Figure 5:**
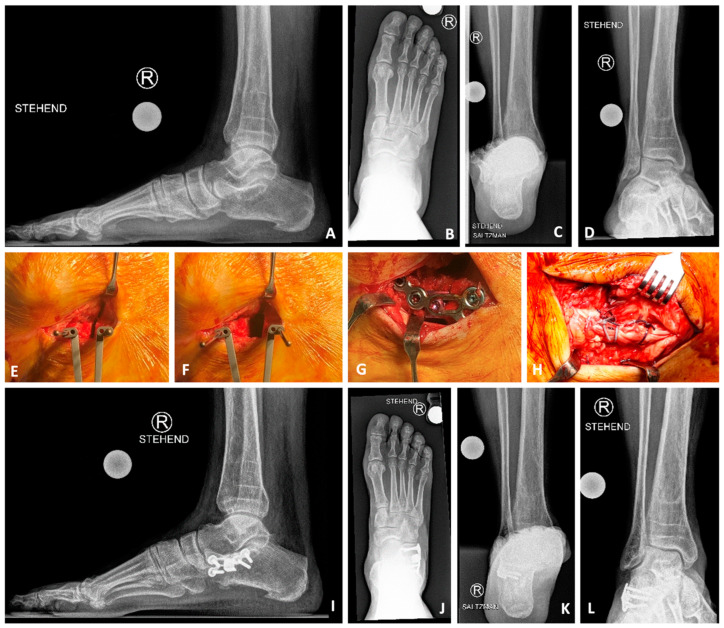
Lateral calcaneal lengthening osteotomy (LCLOT) with anatomical plate and titanium wedge. Sixty-four-year-old male with painful severe pes planovalgus and abductus right foot due to posterior tibial insufficiency with deltoid and spring ligament lesion. Treatment by LCLOT with anatomic Aptus-LCLOT-Plate (Medartis, Basel, Switzerland) and deltoid/spring ligament reconstruction as well as flexor digitorum longus (FDL) to posterior tibial (PT) tendon transfer. (**A**–**D**) preoperative X-rays: lateral foot, AP foot, Saltzman view, mortise view, (**E**–**H**): intraoperative pictures ((**E**) LCLOT with K-wire-forceps, (**F**) open of the LCLOT; (**G**) Aptus-LCLOT-Plate with titanium-wedge (Medartis, Basel, Switzerland) and spongiotic iliac-crest-autograft; (**H**) medial soft tissues reconstruction); (**I**–**L**) postoperative X-rays: 6 months postoperative with three-dimensional improvement of foot position and painfree patient.

The patient is positioned supine. A tight tourniquet is inflated at 250 mmHg. A sinus tarsi approach is then performed. Care must be taken not to injure the peroneal tendons and the sural nerve. The sinus tarsi is exposed. A Hohmann retractor is positioned inside the sinus tarsi, ahead/distal of the interosseous ligament footprint, in order to prevent damage to the ligament and therefore destabilize the subtalar joint. The second Hohmann retractor is positioned beneath the calcaneus, taking away the peroneal tendons/soft tissues and exposing the calcaneus neck completely (Figure 2A). The osteotomy is then performed perpendicular in the space between the posterior and anteromedial facets of the subtalar joint, without breaking the medial calcaneal cortex if possible. Two K-wires are placed on each side of the osteotomy, and, with the help of a K-wire-spreader, the osteotomy is opened till enough three-dimensional correction of the foot is achieved (Figure 2B). This procedure is capable of a tri-planar correction of the foot: Hindfoot varisation/medialization, forefoot/foot adduction, and medial arch elevation are contemporarily achieved (Figure 3). Once the right amount of correction has been clinically assessed, an iliac crest tricortical bone graft is harvested and inserted into the LCLOT as open wedge graft. An allograft or a titanium-wedge are also possible fillers of the LCLOT. The graft is held in position by a 5.0 mm or 4.0 CCS screw depending on the foot size or an LCLOT-Aptus-Plate (CCS Screw/LCLOT Aptus Plate, Medartis, Basel Switzerland) (Figure 4 and Figure 5).

The LCLOT at the floor of the sinus tarsi is the authors’ choice. Performing this procedure at the sinus tarsi has the greatest tri-planar correction of the foot and ankle, achieving the correction of the flatfoot/PCFD by a single osteotomy. Cutting just distal to the interosseous ligament is advisable for preventing subtalar instability. In the opinion of the authors, one screw is sufficient to hold the graft, even though in large LCLOT corrections or severely osteoporotic patients, a LCLOT plate with titanium wedge can be used to ensure a more stable fixation.

The surgeon must keep in mind that severe flatfeet may have additional deformities that might need to be addressed surgically as well, such as forefoot supination (Cotton osteotomy or Lapicotton arthrodesis), hindfoot residual valgus (medial sliding calcaneal osteotomy), distal tibia deformities (supramalleolar osteotomy), medial soft tissues surgeries (PTT debridement, spring repair or reconstruction, FDL transfer or tenodesis) and gastrocnemius recession [4,71].

Postoperatively, the patient is advised to wear a walker with crutches and partially weight bear (15 kg) for 6 weeks. Physiotherapy initially focused on ROM exercises without load. Proprioception, gait and strengthening exercises are progressively allowed after 6 weeks. The patient should be informed that it is common to face an adaptation phase by 3 months after surgery, where pain and swelling could increase again. Although it is important to rule out complications such as nonunion, infection and CPRS, most commonly this is due to the three-month adaptation phase: a physiological transition phase in which the foot passes through a second peak of healing inflammation, adapting to the normal load of the foot and ankle [69].

## 10. Discussion

Calcaneal osteotomies are useful bony corrective procedures for flexible hindfoot pathologies [13,41]. The idea to lengthen the lateral calcaneus/foot column is based on the concept of relative shortening of the lateral column in flatfoot deformity, implicating in talonavicular subluxation and forefoot/foot abduction [34,61]. Several techniques have been described for this purpose and they vary on the osteotomy location, graft usage and type of fixation (results were summarized in Table 2). Short- and midterm results are available in the literature to support any of those techniques.

The first technique closed to the CC-joint was described by Evans 1975, but concerns with lateral CC-joint overload and pain arose [14,35,37]. The fact that most of the population has a conjoined anterior and middle facet also raises concern about potential articular damage with this procedure [47]. A more proximal osteotomy could increase the correction power with a shorter graft and decrease the risks of lateral column overload. Using the space between the posterior and anteromedial facets the lateral calcaneal lengthening osteotomy (LCLOT, sinus tarsi osteotomy) diminishes the chance of articular damage when compared to the Evans osteotomy as described by Ettinger [46]. The z-shaped step-cut calcaneal osteotomy relies on a more complex osteotomy but could provide theoretically better hindfoot valgus correction [49,50]. As many surgeons perform the LCLOT, but call it Evans osteotomy, for comparison of clinical outcome studies, surgeons shall difference between the LCLOT and Evans osteotomy.

On the graft topic, autograft and allograft were described with comparable outcomes in the literature [53,54,55,56]. One should evaluate and discuss with the patient the better option for the case. Iliac crest tricortical autografts are a safe, fast healing, and well-known graft, but the morbidity should not be overlooked. Allografts provide good fusion of the osteotomy site, but the risks and costs should be considered [54]. The use of metallic wedges is described with good results, but more studies are needed to support this option instead of auto-/allografts [59,60]. It is important to consider the graft size and shape. Although there is no consensus on the subject, rectangular and larger grafts improve correction but could be associated with more complications, such as lateral overload, stiffness and nonunion [57,61].

Few studies compared the fixation methods. It seems that a fixation method should be used for maintaining correction and improving stability, thus potentially decreasing nonunion [35,63]. Most of the surgeons use a screw for osteotomy and graft fixation, but the conformation, size and number of screws vary. For stronger and more rigid fixation an LCLOT-plate might be favorable; however, future studies need to prove this.

Good functional results have been described with the LCLOT in a retrospective study using the Foot and Ankle Ability Measure (FAAM) Activities of Daily Living (ADL) and FAAM Sports questionnaires. Although an overall relative high average scores were found, a higher body mass index (30 kg/m^2^ or more) was associated with lower scores, while greater preoperative hindfoot valgus deformity was associated with higher scores [72].

## 11. Conclusions

The lateral calcaneal lengthening osteotomy (LCLOT) is a well-established procedure with good results when used to treat patients with PCFD and forefoot/foot abduction. Surgeons should distinguish LCLOT from Evans osteotomy, since different procedures are described to lengthen the lateral column. Good clinical results and high union rates can be expected regardless of the chosen osteotomy, graft or fixation method. Further better quality biomechanical and clinical studies are necessary to guide surgeons and improve even more the results.

## Figures and Tables

**Table 1:** Lateral calcaneal lengthening osteotomy (LCLOT): Indications.

**LCLOT Indications**
Flexible Pes planovalgus et abductus/Flatfoot/Progressive Collapsing Foot Deformity (PCFD), caused by:
Posterior Tibial Tendon Insufficiency Tarsal Coalition Chronic Medial Ankle Insufficiency Accesory Navicular/Os tibiale externum Foot realignment procedure in the setting of Ankle Osteoarthritis/Osteochondral Lesions

**Table 2:** Summary of calcaneal lengthening osteotomy studies. Analysis divided into: study type, osteotomy type, graft choice, type of fixation, number of patients involved and main results.

Study	Study Type	Procedure	Graft/Fixation	No. of Procedures	Results
Ettinger et al. [48]	Retrospective	Cervical and sinus tarsi osteotomies	Autograft/Screw	54	No statistical difference clinically or radiographically.
Saunders et al. [50]	Retrospective	Cervical and z shaped	Autograft (16%) or allograft (84%)/screws	143 (65C/78Z)	Faster healing times and smaller graft on the Z group with minimum 2 years follow up.
Demetracopolous et al. [51]	Retrospective	z shaped	Autograft/Screws	37	All talonavicular parameters improved. Clinical (FAOS and SF 36) improvement.
Moore et al. [58]	Retrospective	Evans	Titanium wedges	34	Radiographic parameters and clinical improvements. No migration, loss of correction or hardware removal.
Gross et al. [59]	Retrospective	Sinus tarsi	Titanium wedges	26	radiographic parameters improvement, 88% had pain relief, 96% achieved union.
Hintermann et al. [45]	Retrospective	Sinus tarsi	Autograft/screws	19	Pain relief, Radiographic parameters improvement and only 1 case of nonunion.
Dolan et al. [53]	Prospective Randomized	Evans	Autograft or allograft/screws	33 (18 allograft/15 autograft)	No nonunions, two patients in the autograft group with donor site at 3 months.
Grier et al. [54]	Retrospective	Evans (18) or CC arthrodesis (33)	Autograft (20) or allograft with PRP (31)/screws	51	Union 70% for autograft and 94% for allograft. More complication and hospital stay for autograft. More cost for allograft.
Vosseler et al. [55]	Retrospective	Evans	Autograft (51) or allograft (75 isolated and 45 associated with BMA)/vary, but mainly single screw (4.0 mm)	126	Rate of nonunion and loss of correction for LCL was not significantly different between allograft and autograft.
Müller et al. [56]	Retrospective	Sinus tarsi	Autograft or allograft/screws	50	Loss of hindfoot alignment and graft incorporation not statistically significant.
